# Towards an Evolved Immersive Experience: Exploring 5G- and Beyond-Enabled Ultra-Low-Latency Communications for Augmented and Virtual Reality

**DOI:** 10.3390/s23073682

**Published:** 2023-04-02

**Authors:** Ananya Hazarika, Mehdi Rahmati

**Affiliations:** Department of Electrical Engineering and Computer Science, Cleveland State University, Cleveland, OH 44115, USA; a.hazarika@vikes.csuohio.edu

**Keywords:** augmented reality and virtual reality, end-to-end latency, ultra-reliable low-latency communications, 5G and Beyond network design

## Abstract

Augmented reality and virtual reality technologies are witnessing an evolutionary change in the 5G and Beyond (5GB) network due to their promising ability to enable an immersive and interactive environment by coupling the virtual world with the real one. However, the requirement of low-latency connectivity, which is defined as the end-to-end delay between the action and the reaction, is very crucial to leverage these technologies for a high-quality immersive experience. This paper provides a comprehensive survey and detailed insight into various advantageous approaches from the hardware and software perspectives, as well as the integration of 5G technology, towards 5GB, in enabling a low-latency environment for AR and VR applications. The contribution of 5GB systems as an outcome of several cutting-edge technologies, such as massive multiple-input, multiple-output (mMIMO) and millimeter wave (mmWave), along with the utilization of artificial intelligence (AI) and machine learning (ML) techniques towards an ultra-low-latency communication system, is also discussed in this paper. The potential of using a visible-light communications (VLC)-guided beam through a learning algorithm for a futuristic, evolved immersive experience of augmented and virtual reality with the ultra-low-latency transmission of multi-sensory tracking information with an optimal scheduling policy is discussed in this paper.

## 1. Introduction

### 1.1. Background and Motivation

With the enormous growth in commercialized fifth-generation (5G) cellular systems and 6G technology on the horizon by 2030 [1], there is an increase in the demand for ultra-reliable low-latency communications (URLLC) [2,3] to provide an end-to-end latency of 1–5 milliseconds or less to replace the traditional connectivity techniques. Moreover, considering the infinite possibilities of augmented/virtual reality (AR/VR) enabled by 5G networks, the maintenance of a millisecond to sub-millisecond latency threshold is of utmost importance to unlock many game-changing technologies in AR/VR applications such as in industrial applications, holographic displays, and the touch Internet, i.e., the Tactile Internet [4], just to name a few. Due to the most-significant characteristics of 5G, namely its multi-radio access technology (multi-RAT) nature, 5G has the capability to cooperate with other radio technologies such as WiFi, a license-free technology deployed up to 200 Mbps [5]. WiFi is being claimed as the most-popular unlicensed wireless technology for providing access to high-speed Internet over wireless local area networks (WLANs), and it is estimated to support 9.5 billion devices for emerging AR/VR applications. The current generation, WiFi 6, technically known as WiFi 802.11ax, performs better due to its higher throughput of reaching 9.6 Gbps. Although 5G provides huge flexibility in deployment by supporting an enormous range of users and services, WiFi is comparatively easier to deploy through wireless routers and access points for full network coverage. Two frequency bands, namely sub-6G with a bandwidth of 100 MHz and mmWave with a bandwidth of 400 MHz, are utilized by 5G networks, whereas three non-overlapping channels with a bandwidth of 60–80 MHz are utilized by WiFi 6 access points to obtain a high performance and immersive environment in AR/VR [6]. Moreover, 5G offers immense advantages compared to WiFi 6, which is limited to a finite bandwidth per user, by providing full end-to-end connectivity due to its combined merits achieved from the mid-band and low-band and an ultra-low latency environment from the high-band in mmWave [7]. The expected theoretical latency of WiFi 6 stands at 36 ms, whereas the desired theoretical latency for 5G is within the range of 6–9 ms [6].

The end-to-end latency requirements for an AR/VR system include an interactive remote environment with a latency often ranging between 40ms and 300ms, thus providing richer visual content with a higher resolution, a higher frame rate, a high dynamic range (HDR), and 360° spherical six-degree-of-freedom (DoF) content [8]. Though enhanced Mobile Broadband (eMBB) in 5G plays a pivotal role in supporting a bandwidth that is higher than that of 4G, it is capable of offering much less improvement to low-latency without compromising on the bandwidth [9]. Therefore, this issue demands the requirement of ultra-wideband (UWB) wireless technologies [10] in enabling a low-latency system for optimized use in high-quality audio and emerging AR/VR applications by allowing timely transmission with a higher throughput. This UWB system requires an appropriate antenna design to cover both traditional LTE and sub-6G bands simultaneously [11]. Such a design requires an interface covering both line-of-sight (LOS) and non-LOS (NLOS) scenarios [12] coupled with novel hardware designs for supporting the bandwidth requirements of 6G networks by utilizing radio-frequency (RF) micro-electromechanical-system (MEMS)-empowered terahertz switches to handle a wider range of spectrum with a reduced response time [13].

**Figure 1:**
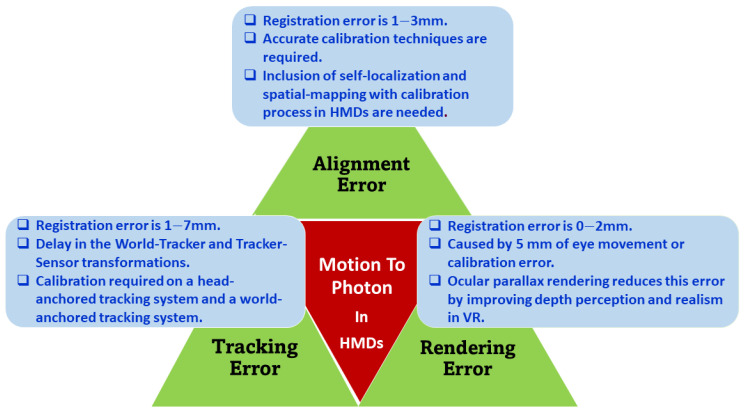
The block diagram shows the main categories and various types of errors associated with MTP latency in AR and VR HMDs.

There are several factors responsible for the association of delays with the simulation environment and the AR/VR devices while presenting an enhanced view of the surroundings to the users in the virtual world. The low end-to-end delay in AR/VR devices arises due to the steps involved in sensor sampling, data processing and fusion, image rendering and encoding, transmission and decoding, and displaying of each frame. These are some of the main causes for the occurrence of the motion-to-photon (MTP) latency, as shown in Figure 1. Therefore, innovative network designs, technologies, methods, and devices must be considered to improve the traditional AR/VR systems by providing an immersive seamless VR experience with extremely low end-to-end latency. However, it is required to keep the MTP latency under 20ms as the VR cloud services are extremely sensitive to low latency, which may cause dizziness for the users. It is challenging to maintain a low MTP latency as VR terminals have to undergo a serial process of motion capture, logic computing, picture rendering, and screen display. Hence, despite the extensive advances in AR/VR technologies, MTP latency [14] acts as the greatest barrier by holding back AR/VR adoption from proving a fully immersive experience and offers limitations in the field of view due to occlusion, as well as poor display quality. A very minimal MTP latency of less than 7ms for head-mounted display (HMD) users is required to avoid having an unrealistic experience of the virtual world [15]. It has been proven [16] that a latency of 5ms or less for both AR and VR should be experienced to have a decent effect on users without causing any motion sickness. When the data from the HMDs fail to register spatially and temporally with the user’s view of the surroundings, an effect called misregistration occurs. This phenomenon is a result of a mismatch in measuring the time consistency in tracking data displayed on the HMD considering the location and orientation of the controllers [17]. Figure 1 presents a brief description of the various types of errors arising from MTP latency in AR/VR devices. Three types of major errors, namely alignment, tracking, and rendering error, arising from MTP in HMDs along with their quantity and the technique needed to mitigate these errors are mentioned in this figure. Some hardware devices, such as optical see-through (OST) virtual headsets and simulator systems, are commonly used to translate the virtual content into the real world. However, they can also be affected by latency in the form of misregistration. Several techniques, such as time warping [17] or post-render warping [18], have been proven to improve the average quality, as well as the consistency of latency on both the display and the rendering engine.

With the unprecedented growth of VR HMDs following the announcement of the Meta Oculus [15], there still exists a gap between the end-to-end delay and a reliable wireless connection in AR/VR technology. As the latency measured in the Oculus Quest 2 is around 50–70 ms [19], people usually resort to Air Link, which is an external USB cable, to minimize the latency by 10 ms. The latency for hand gestures while playing games, even after using Air Link, remains at 29 ms, making the delay seem pretty high.

### 1.2. Vision and Insight

To meet the latency requirements of various AR and VR applications, a comprehensive discussion and quantitative analysis of the specifications are required. Novel communication designs with ultra-high speed, low power, and ultra-low latency specifications should be envisioned and developed. This paper discusses existing technologies, as well as several breakthrough solutions and advancements in ultra-low-latency communications in the 5G and Beyond era. Technologies such as WiFi 7, mobile edge computing (MEC), and the Tactile Internet will unlock new possibilities in remote control, mobility, automation, and industrial control, motivating communications service providers to further upgrade users’ experiences in real-time use-cases such as cloud gaming and AR/VR applications. Using such wireless technologies for network design, low-latency connectivity can also be achieved by focusing on finding an optimal end-to-end packet transmission path from transmitter’s Packet Data Convergence Protocol (PDCP) to the receiver’s PDCP from the device all the way to the application, wherever it is located (e.g., in a data center or cloud provider), and back. This leads to a necessity to meet all the requirements of an optimal message transmission [20], technologies, and the topology of the overall network architecture. Additionally, these technologies require an intelligent wireless network design by utilizing the mmWave-based technologies to support their requirements of an ultra-reliable and low-latency connection for delivering a realistic and seamless spatially aware experience to the user. Several technological challenges, such as video delivery through the mobile network and balancing the trade-offs with the gesture/head movement of AR/VR headsets, contribute to the delay in VR/AR technologies. Based on these requirements, this visionary paper focuses on providing an insight into the usage of several hardware and software requirements with the integration of 5G technology towards the upcoming 5GB networks. We illustrate a learning-based framework for enabling an evolved experience of using AR/VR with a focus on ultra-low-latency communications.

### 1.3. Paper Overview

**Figure 2:**
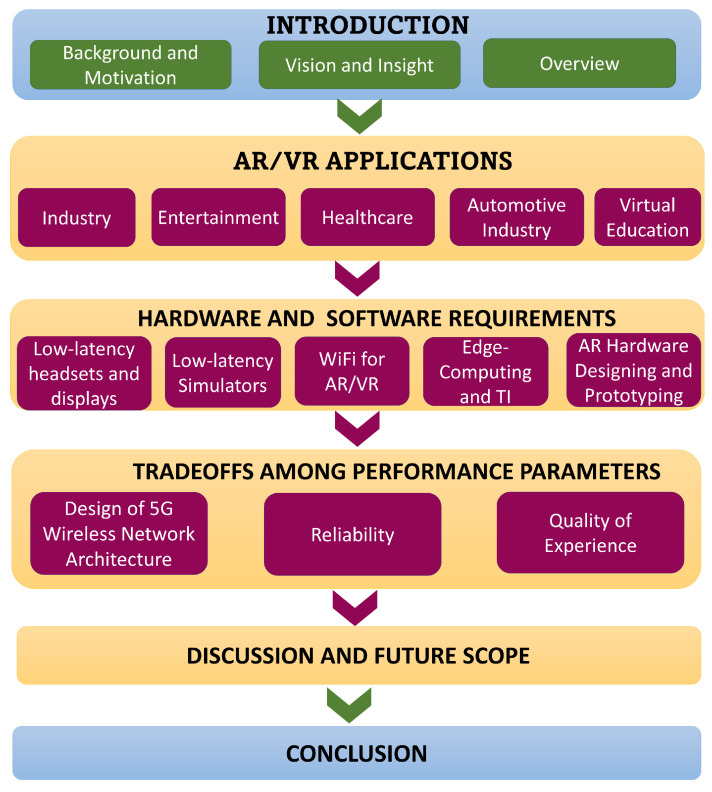
Outline of the paper.

The remainder of this paper is organized as follows. In Section 2, we discuss the AR/VR applications in different verticals. In Section 3, we explore the requirements for ultra-low latency from the AR/VR software and hardware perspectives. In Section 4, we present the various trade-offs associated with the performance parameters to ensure a reliable and low-latency communication. In Section 5, we provide a brief discussion on the challenges and the possible directions for future research. Finally, in Section 6, we conclude the paper. Figure 2 depicts the organization and overview of the paper. The acronyms used in the paper are listed in Table 1 along with their respective definitions.

## 2. AR/VR Applications

**Figure 3:**
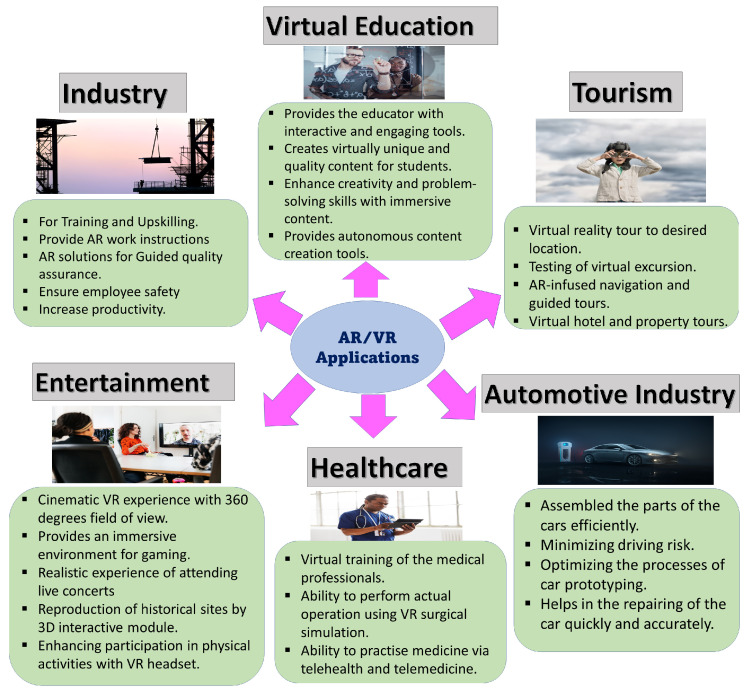
The major use cases of AR/VR.

In this section, we review the main categories of applications that are using AR and VR technology as summarized in Figure 3. In Section 2.1, we highlight the benefits gained by Industry 4.0 from the implementation of virtual reality and augmented reality. In Section 2.2, we describe the ways in which AR and VR are transforming the entertainment industry by making it more immersive and engaging for the users. Section 2.3 discusses several possibilities for the use of AR/VR technology in healthcare and its evolution. In Section 2.4, the contribution of AR and VR to the automotive industry by creating a safe environment for the driver and making the assembly of cars more efficient is discussed. Section 2.5 presents the ways in which the increasing demand for or awareness of AR/VR technologies is benefiting the travel and tourism industry. In Section 2.6, the applications of virtual education to provide personalized learning or regulated environments for the learners in creative, innovative, and fun ways are highlighted.

### 2.1. Industry 4.0

With the need for a new approach to the modern economy, the concept of Industry 4.0 has been introduced for innovation and technological development in the next industrial revolution by utilizing machines/robots to mimic the work of humans [21]. Industry 4.0 plays a pivotal role in building modern industries with the integration of several disruptive technologies such as the Internet of Things (IoT), machine learning, computing platforms, cyber-security, robotics, simulation, additive manufacturing [22,23], and AR/VR. The main goals of Industry 4.0 are to apply advanced IT-based technology for mass customization of manufactured products and to foster the adaptive flexibility of the production chain by tracking the growth of parts and products by applying the human–machine interaction (HMI) paradigm [24]. Industry 4.0 makes use of IoT-enabled technology for optimized production in smart factories and develops business models of interaction in the value chain. The technologyperforms the role of a skilled operator such as a robot aided by a machine.

AR/VR plays an important role in industrial innovations and contributes to their increased growth by bridging the existing gaps between the physical and virtual spaces by allowing people to exist in multiple different realities, mediums, and forms. Recently, investors have accelerated their interest in the AR/VR industry, and it is predicted that the industry will grow by more than USD 125 billion by 2024 [25]. Initially, AR and VR were utilized by retailers for the promotion of their products and services to customers. With the advancement of software algorithms and cutting-edge hardware in AR/VR, they are widely used in industry as a primary resource in supervising and training industry workers to be efficient enough to tackle challenging tasks [26]. In AR, viewers get to experience some prominent scenes of the real world by overlaying the virtual things on real-world objects. AR/VR plays a pivotal role in production growth by enabling a real-time interactive visualization of production line operations and constantly supervising various phenomena affecting them. With the help of extreme computing power and low latency in sensor data provision, processing, analysis, and visual rendering, AR/VR has been able to overcome all the shortcomings of Industry 4.0 [27]. Some of the advantages of AR and VR’s usage in Industry 4.0 include their capability to generate a virtual 3D character or the user’s avatar by recording his/her physical movements. AR/VR can contribute to the maintenance and repair process by helping workers with the generation of a multimedia-based instruction manual or handbook when they are engaged in some complicated and time-consuming tasks. AR/VR has the capability to increase the work’s efficiency and reduce the cognitive load [28] of the workers by assisting them with the display of procedural information using HMDs. Recently, Industry 5.0 has been introduced to complement the Industry 4.0 paradigm with extensive advancement in research for innovative transformation in a human-centric, sustainable, and resilient manner. Industry 5.0 [29] will witness a huge shift from a technology-oriented approach to a human-centric and society-centric approach to improve the decision-making process.

### 2.2. Entertainment

AR and VR provide maximum benefit in the entertainment industry with their applications based on improving the gaming experience and enhancing cinema, events, shows, and museums, hence attracting more customers. The gaming industry is investing a massive amount to deploy virtual environments in entertainment as the gaming market size is expected to grow at a compound annual growth rate of 30.2% from 2020 to 2027, as provided by Grand View Research [30]. The use of VR and AR creates an immersive environment for the gaming audience. We can consider the example of VR gaming in Ace Combat 7, where the game utilizes detailed aircraft and photorealistic scenery to provide a realistic simulation experience to the players as if they were really flying in a fighter jet [31]. AR/VR provides the realistic experience of attending live concerts while saving time by building an audience with the help of the Metaverse [32]. Artists can develop a three-dimensional virtual environment to showcase their work or ideas to the audience. The audience can experience nostalgic and insightful histories and culture without visiting a museum with the 3D interactive modules and the VR space provided by the AR/VR technologies [33].

The authors in [34] provided an explanation of a novel framework known as Harmonize to show the applications based on shared environments (SEs) for AR and VR users. Harmonize was an innovative app built with the intention of utilizing the advantages of a free and powerful engine used for animation and scene creation. In order to test and evaluate the validity of Harmonize, an immersive game was developed to offer a similar gameplay experience to both AR and VR users. The headset HTC Vive, which was made in collaboration with PC games giant Valve, was packed with 70 sensors to offer 360-degree head-tracking, as well as a 90 Hz refresh rate to the reduce latency that is responsible for causing motion sickness. Due to this issue, VR headsets with higher refresh rates are required. There is a high possibility that VR headsets with high data rates could result in a low screen resolution and low headset refresh rates. Therefore, the need for intensive computational capability and a massive communication bandwidth with ultra-low latency to transmit high-resolution/high-frame-rate videos to VR headsets arises. These demands for a massive communication bandwidth with ultra-low latency can be fulfilled with the usage of edge caching and mobile edge computing [35], which provide content and computing resources to the users with reduced latency, which are not supported by current wireless mobile networks.

### 2.3. Healthcare

AR/VR is gaining popularity in the healthcare sector, and the market for AR/VR in healthcare isexpected to reach nearly USD 9.7 billion in value in the next 5 years [36]. The usage of HMDs, such as goggles or headsets, enables users to interact with a computer-generated environment responsible for stimulating multiple sensory modalities, including visual, auditory, or haptic experiences [37]. The introduction of an entirely new perspective in AR/VR in the practice of medicine has enabled a systematic strategy for training physicians and other medical professionals. Medical professionals highly benefit from the AR/VR training procedure because it augments their ability to practice medicine through telehealth and telemedicine. The trainee can gain surgical experience using a VR surgical simulation system [37,38] that generates the scenario of performing an actual operation and is highly proven to reduce the error during an actual operation in the future. With the steady growth of three-dimensional avatars in the VR industry, they can be used to represent three-dimensional stereoscopic visual effects to gain an understanding of the positional relationship between objects and simulations. They can simulate the process of intravenous injection to deliver knowledge related to human anatomy [39] to medical professionals or students. Recently, the Autism Glass Project has been initiated by the Medical School of Stanford University, which uses the Google Glass AR technology to help interpret the emotions of children with autism to help them develop normal social relationships [40]. While performing surgery or during any severe injuries, pain can be controlled by a less complicated adjunctive pain control mechanism performed in the clinic on an outpatient basis provided by VR [41]. These AR/VR techniques rely on cognitive–behavioral techniques by generating VR environments to relieve pain [42].

### 2.4. Automotive Industry

AR/VR technology has a huge impact on the entire end-to-end process involved in the design, production, sale, and even marketing of automobiles. Moreover, the usage of the 5G network makes the adoption of these futuristic AR/VR technologies for high-speed data transfers through smartphones and Internet penetration with very low latency easier. The classical robotics methods used for risk assessment in road safety have been dominated by the usage of AR/VR technologies in autonomous vehicles to enhance road safety and improve the general driving experience [43]. Three types of AR displays, namely heads up displays (HUDs), HMDs, and heads down displays (HDDs), have been deployed for car navigation systems to guide the users with navigation information [44]. To develop autonomous vehicles (AVs), an extensive amount of training and testing of various AI approaches is required, which are expensive and time-consuming. AR/VR has been considered as the most-innovative tool to overcome these limitations due to the requirement of different simulation models to generate extensive data and test the developed AI algorithms. Their models replace existing simulation-based research on AV platforms such as MATLAB and CarSim for the evaluation human behaviors and road environments to estimate the potential safety issues of AVs [45,46]. With the advancement of VR devices, multiple implementations of HMDs were shown in [34,47,48]. To imitate the human driver’s decision-making when driving an AV, reinforcement learning (RL) has been used to evaluate risky scenarios by finding the optimized steering angles and velocity to reduce the risk of collision.

### 2.5. Travel and Tourism

As the outbreak of COVID-19 has resulted in a huge loss in revenue for the tourism industry, leaders are resorting to AR/VR devices as a powerful tool for boosting the post-pandemic tourism industry [49]. With the rise in demand for travel and hospitality services, travel companies are willing to invest in AR/VR technology to provide an immersive experience to their customers. VR has been proposed as a substitution for travel, which implies that it can be used as a replacement for physically visiting certain destinations, thus providing numerous benefits [50]. AR/VR technologies generate a realistic, easy, and detailed navigation of the tourism places and enable travelers to experience a bird’s-eye view of their desired destination [51]. VR can be used as an effective tool in planning a trip by allowing the tourist to communicate with other members via social media apps to gain insights into the destination through feedback from other travelers regarding their previous experiences. An AR-/VR-based application known as VirtualCruiseTour [52] is a technology dedicated to promoting shore excursions by allowing future customers to experience virtual 360° pictures or videos either in an immersive VR or non-immersive mode of their sites of interest while on a cruise. This technology has an AR module that augments a map to help the customers with the cruise route and by locating their current position and the next stop at the desired location. A marker-based approach has been used to create this AR module, where the marker (typically an ad hoc 2D image) is in the camera frame to locate the virtual content superimposed on the real world. According to Accord Marketing [53], a marketing agency, VR tourism has been rated as the third-most-popular virtual activity after virtual museum visits and virtual gaming. Many luxury hotels and properties have the option of virtual tours of hotel rooms and luxury suites so that customers can view these before making their purchase.

### 2.6. Virtual Education

AR and VR have gained popularity by providing students an enhanced authentic classroom experience equipped with instructions from the educator. Utilizing AR/VR technologies in classrooms has been proven to be successful in engaging students with truly unique and quality content with 360-degree views. VR tools are used to supplement the materials of the educators by helping them enhance classroom instruction by overcoming discipline restrictions and offering the students an immersive learning experience. CAVE, an immersive virtual reality environment, is considered a suitable choice for classrooms as it can be easily used inside a room whose walls, ceilings, and floors may have projection screens [54]. CAVE has a significantly lower price compared to the other HMDs and makes it easier for the teachers to assist the students in performing experiments [55]. A novel framework known as ScoolAR [56] was designed as an autonomous content creation tool for AR/VR applications by allowing the teachers to plan structured and tailor-made didactic proposals involving the students and also to avoid content that is misleading in nature. A mobile augmented reality (MAR) game named Alien Contact was designed by Dunleavy, Dede, and Mitchell for teaching mathematics, language, arts, and scientific literacy to middle and high school students. An MAR game called Explore! [57] was presented by Ardito and others to support middle school students in the exploration of archaeological sites in Italy. Google Expeditions Footnote1 [58] is a virtual application mainly used for educational purposes, which allows the users to be a part of several virtual visits to the most-evocative locations around the world including virtual travel to the depths of the oceans and space. Recently, the Air Force has introduced a virtual-reality-based flight simulator [59] designed to train pilots to have adequate flying skills in a safe environment. This flight simulator provides an immersive and natural experience for the pilot of a real aircraft by replicating the movements of a real-world control joystick in a cockpit.

## 3. AR/VR Hardware and Software Requirements

**Figure 4:**
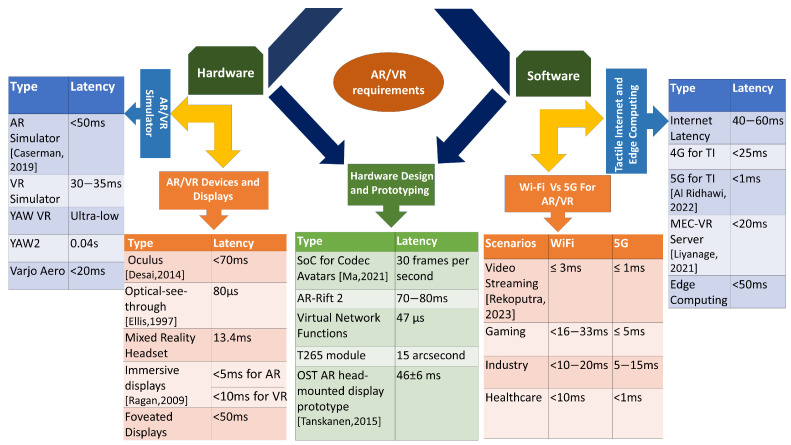
This figure details the requirements for various types of latencies associated with AR/VR technologies from the hardware, software, and design perspectives in order to enable a low-latency connection in an immersive environment.

In this section, we review the major requirements for enabling a low-latency immersive experience from the hardware and software perspective of AR/VR technologies, as shown in Figure 4. In Section 3.1, we describe the requirements of AR/VR displays and headsets to enable a low-latency connection. Section 3.2 provides a brief idea about how to satisfy the low-latency requirements of AR/VR HMDs. In Section 3.3, we highlight the design and prototyping of several components of hardware integrated with AR/VR devices to reduce the latency of the system. Section 3.4 shows the advantages of Next-Gen WiFi in ensuring low-latency communication for AR/VR applications. Section 3.5 presents the low-latency requirements of powerful technologies such as edge computing and the Tactile Internet to support 5G and the IoT in different emerging AR/VR applications.

### 3.1. Low-Latency Displays and Headsets for AR/VR

The AR/VR industry has witnessed an evolution with the release of several consumer-grade VR headsets (e.g., Oculus Rift CV11, HTCVive2, and PlayStation VR3) in recent years [60]. These devices are responsible for linking the users with an environment created by the coexistence of the real and virtual world in real-time. They require a registration [61] that is both spatial and temporal in nature with very few errors so as to maintain this coexistence of the real world with the virtual one. However, some errors [62] occur in the registration due to system latency, which results in the distraction of the users due to the lag in the virtual imagery arriving at the intended position. The system latency is caused by the change in the imagery in response to the user’s movement while using the VR headset. When the rendering of the imagery occurs frame by frame with the transmission of the data to the display in the conventional manner, the registration errors cause latency. To minimize the system latency caused by waiting for the vertical synchronization, Nvidia introduced a head-worn display system known as G-Sync${}^{\mathrm{TM}}$, where the delay can be reduced with the maximization of the input responses by allowing the display to accept the frames immediately after being rendered by a graphics processing unit (GPU) [63].

Motion-to-photon (MTP) latency is the difference between the time of the motion captured by the headset sensor and the time of the appearance of the image on the headset’s display. Long MTP latency can cause various side-effects for users, such as nausea or motion sickness. Inertial measurement unit (IMU) sensors play an active role in sampling the motion data consisting of the user’s pose information, which will be later transmitted to the rendering engine to generate a framebuffer [64]. A time lag is observed when the framebuffer is sent to the headset to display the rendered imagery. Therefore, a post-render warping approach has been introduced [65] for this type of headset to optimize the latency. This technique uses homography transformation to transform an image captured at a specific camera pose into an image at a new camera pose. The addition of a post-rendering stage with the usage of a light-weight, low-cost, optical-see-through (OST) HMD has also been shown to be highly beneficial to overcome the temporal inconsistency of registration [61]. This leads to an updated rendered imagery with the most-recent data based on head tracking. This display provides an average end-to-end latency of 80 μs and can present binary frames to the user up to 16kHz. The processing of post-render warping is performed by a field programmable gate array (FPGA) for just-in-time tracking of the updates by utilizing a novel pseudo-random pulse density modulation (PDM) [61] technique for the conversion of binary pixels into a perceived grayscale format. A tracking system to report the updates frequently can be utilized to reduce the perceived latency to the sum of that tracking latency for about 80 μs to 124 μs of display latency. In addition to the OST display technology for post-render warping, an open-source mixed reality headset, supporting both virtual and augmented reality applications [66], provides a better MTP latency of 13.4ms on average and delivers an immersive experience to the user by using the post-render warping technique along with a 240Hz digital light projector (DLP). The Xilinx Ultrascale+ ZCU102 System-on-Chip [67] has been used to prototype the hardware architecture, and the post-render warping accelerators are processed by the FPGA of the ZCU102 to design the system of the headset. The liquid crystal displays (LCDs) of headsets are being replaced with the Texas Instruments DLPs as the projectors for AR/VR displays.

A new strategy to determine total system latency was introduced in [68] to measure the cognitive latency using video see-through devices when it becomes hard to instrument the input to measure its corresponding output signals. It was verified in [68] that the delay of humans performing a rapid motor task using different video see-through devices and also in front of a computer would correspond to the system latency. A hardware instrumentation-based technique for benchmarking has been used to measure some types of latency. Such a new form of latency measurement through human cognitive performance can be reliable and comparable to hardware-instrumentation-based measurement. A standardized canonical test was introduced to perform cognitive latency tests to measure multiple latencies [69] successfully, which included the photon-to-photon latency and the tracking latency. The test was performed on four HMDs, where the first device was an ad hoc system, known as Prism, and the rest of them were the Oculus Quest, which is an untethered VR system, the Oculus Rift S, and the Valve Index, which are both tethered VR HMDs. The Prism ad hoc system [70] with a pair of color cameras connected to an Acer Windows Mixed Reality device with a display resolution of 1440 × 1440 per eye at 90Hz was designed to generate a close-to-optimal see-through low-latency AR experience and minimize the amount of re-projection between the cameras and the display. The pair of cameras attached to the Prism device had sensors configured to capture video at 90Hz at a resolution of 1704 × 1440 pixels, which were custom-built using Omni Vision’s 4689 sensors. The latency of the system was measured with a hardware-instrumentation-based method based on a sub-millisecond accuracy clock. To measure cognitive latency, a rapid task similar to [71] was performed several times until the completion of 10 error-free trials for each HMD. It was found that the performance of the users directly impacted the latency, as the standard deviation of the error had a direct impact on the users’ behavior due to the occurrence of an error arising from releasing a button too early.

### 3.2. Designing Low-Latency AR/VR Simulators

The selection of an optimal simulation platform plays a pivotal role the analysis and study of different types of latency. For the modeling of a simulation platform, adequate training is required by interacting with various complicated or costly scenarios of real outdoor environments. The simulator utilizes VR to simulate a complete AR system, which reduces the latency of the system. The simulator creates an arrangement responsible for precisely controlling the registration of virtual objects and allowing testing for the presence of registration error [72]. An approach was introduced in [72] for the evaluation of a perfect registration using VR systems through complete control to isolate and independently manipulate different types of registration errors for a low-latency system. An approach for confirming the relation between latencies obtained from the investigation of registration errors and task performance was given by Ellis [66] using an actual AR system. The setup consisted of an augmented virtual tube along with the rendering of an ARToolKit marker on a table in a simulated real-world environment. They used a virtual ring that was superimposedon another marker by attaching it to the end of a stick, and they moved the ring from one end of the tube to the other while keeping the tube inside the ring to investigate the relationship with the latency based on the task performed. Using this approach, the registration errors causing latency and jitter were controlled. Latency errors of 0, 176, 352, and 528 ms were simulated by controlling the frame delays for the simulated virtual ring experiment. In [73], the effects of different types of latency obtained from representative simulations for AR training on various tasks were studied. A simulation setup was prepared for both the real environment, as well as all AR augmentations in a high-fidelity VR environment to isolate the effects of different latency parameters on task performance.

### 3.3. AR/VR Hardware: Designing and Prototyping

AR/VR utilizes 3D lifelike virtual avatars known as codec avatars [74] to mimic human behavior in the virtual world. They are implemented using two main building blocks, i.e., encoding and decoding [75,76]. These two blocks work simultaneously to virtually represent faces in a realistic fashion. Firstly, the encoding captures the facial expression data of one user for continuous transmission to the other user, and then, the decoding is performed by rendering a lifelike avatar on the other user’s headset. However, the need to achieve high frame rates with frequent updating of the status in the encoding and decoding blocks to have a low latency for the codec avatars for realistic interactions arises. As a result, these mobile AR/VR devices should possess the capability to provide high visual computing requirements to reach a target frames per second (fps). To overcome these limitations of the workload of the codec avatars on mobile AR/VR devices, a silicon solution has been designed to achieve the requirements of a low-latency system [77].

A small-scale system-on-a-chip (SOC) prototype was presented in [78] for low-latency energy-efficient performance and to enable eye gaze extraction of the codec avatar model, which used a test chip, fabricated in 7nm technology, featuring a neural network (NN) accelerator consisting of a 1024 multiply–accumulate (MAC) array, a 2 MB on-chip SRAM, and a 32-bit RISC-V CPU. This VR-based prototype system used a low-power deep neural network (DNN) accelerator test chip to offload some of the workload. To meet the challenging mobile AR/VR SoC specifications for a codec avatar demonstration, a convolutional-NN (CNN)-based eye gaze extraction model was utilized to mitigate the system-level energy and latency costs for the entire model to fit on the chip. This approach enabled the prototype SoC to achieve 30 fps, satisfying the requirements for a system with low latency and low power consumption. To estimate the 3D pose, i.e., translation and orientation in a 6-DoF pose, of the observer, visual–inertial odometry (VIO) was utilized to measure the motions from the images of the camera(s) and linear acceleration and angular velocity from the IMUs. A lower pose update latency was required for the estimation of the 6-DoF pose of the HMD to achieve a latency of less than 20 ms for a motion-sickness-free immersive experience. The noisy data obtained from the camera were analyzed by a maximum likelihood estimator to estimate the system dynamics or the state in a typical VIO processing pipeline [79]. There are some VIO systems whose functioning is similar to an extended Kalman filter (EKF) [80] to update their states and decide the best pose estimate available. The best pose is decided immediately upon receiving the sensory inputs, including the image frame or IMU sample, without waiting for an optimal solution via local or global optimization based on past estimates. The acceleration of EKF-based monocular VIO [81] was modeled in [79], by a flexible, scalable, and power-efficient micro-architecture. This enabled direct processing of IMU samples without pre-integration to obtain an ultra-low latency and accurate instantaneous pose updates. This micro-architecture was designed to be adaptive and easy to integrate with a host CPU or micro-controller, enabling software-based configuration control while retaining programmability. This hardware architecture, denoted as VIO HWA [79], was integrated in a typical embedded vision SoC in which the identified acceleration candidates were partitioned into the VIO HWA as specialized modules interfacing an on-chip Shared L2 SRAM (SL2) sub-system. They utilized some intelligent pre-fetching techniques to make all the modules of the micro-architecture flexible and tolerant to the data fetch latency by leveraging decoupling first-in, first-out (FIFO) in order to hide the latency of data access to SL2. There was a feature detection module that implemented FAST9 feature detection with a 16 pixels per cycle throughput [82] using a novel 2D sliding window pixel buffer. The functionality of the feature sorter module was to generate a list of a configurable N-number of the strongest features by sorting the detected feature points according to their strength. The feature tracker module also performed the task of prediction-based tracking, where the predicted positions of feature points were calculated using the most-recent EKF state. By using the newest EKF center to define the search center of a configurable search window, the best-matching image patch center was computed as the tracking result from the normalized cross-correlation (NCC) score over the 7 × 7 image patch. As a result, the latency due to tracking was directly impacted by vision path update latency. Therefore, the feature tracker was considered a reliable choice to reduce the latency as it used the high-throughput FAST9 corner point selection logic for the detection of candidate FAST9 corner points within the search window to prune down the number of NCC evaluations. The NCC computation was achieved by an unbiased rounding method on all the intermediate subtraction, multiplication, and accumulation of integer pixel values with the aim to maintain a fully integer-based processing pipeline for the entire NCC computation supporting a fully pipelined NCC computation logic with only a 16 cycle latency and 3 cycles per NCC throughput.

### 3.4. WiFi for AR/VR

Both 5G and the WiFi 6 enhancements are acting as potential technologies to satisfy the market demands with their increased throughput and quality of service (QoS) requirements. The limitations of capacity and coverage occurring in 5G technology due to the lack of resources can be overcome by utilizing the free unlicensed spectrum in WiFi technology. Orthogonal frequency-division multiplexing (OFDM) modulation facilitates the coexistence of both technologies to enable higher speeds, high capacity, and low latency for enhanced QoS performances [83]. WiFi is a powerful wireless communication technology for connectivity in AR/VR applications. Qualcomm announced new WiFi 6E wireless chips, known as the FastConnect 6900 and 6700 chips [84], designed for mobile devices. They support VR-class latency for streaming VR over WiFi. These WiFi 6E Qualcomm chips are an extension of the previous WiFi 6 (based on 802.11ax), open up more channels and bandwidth up to 3.6 Gbps, and provide a latency of less than 3 ms. Innovative applications such as wireless backhauling, AR/VR, 8K video streaming, and sensing are enabled by 5GB communications focusing on the performance analyses of wireless protocols and standards. With the progress of IEEE 802.11be, expected to be marketed as WiFi 7 by the WiFi Alliance, the IEEE standard association is investigating the possible upgrades for the “Beyond be” or “Next-Gen WiFi”, potentially touted as WiFi 8. WiFi 7, known for its full-duplex nature, aims at overcoming the challenges faced by the half-duplex communication system in WiFi 6 and earlier generations of WiFi. WiFi 7 has many advantages such as the utilization of available resources for increased flexibility in dense deployments, the usage of hybrid automatic repeat request (HARQ) in enabling a reliable and low-latency transmission, and the addition of time-sensitive networking (TSN) for a jitter-free, seamless AR/VR environment [85]. WiFi 7 is projected to have an extremely high throughput by supporting a higher peak data rate of 30 Gbps per AP, which is four-times faster than WiFi 6 [9]. An improvement beyond WiFi 7’s technological advancements is required for more innovative solutions to reduce latency and support data rate demands for AR/VR applications apart from the usage of WiFi 7 for the Internet of Things (IoT), high-resolution video streaming, low-latency wireless services, etc. Artificial intelligence (AI) and machine learning (ML) are among the fundamental solutions for Next-Gen WiFi to ensure the ultra-reliability and low latency of the service.

A task group to enable AR/VR applications known as IEEE 802.11bf [86] has been introduced by the IEEE as an extension to the current IEEE 802.11ay for WLAN sensing with high throughput functionalities and centimeter-level sensing resolution. IEEE 802.11bf defines WLAN sensing technology as the WiFi signals used for performing sensing tasks by making use of the wireless signals received from WLAN-sensing-capable stations. The obtained wireless signals are then examined to determine the features (e.g., range, velocity, angular, motion, presence or proximity, gesture) of the intended targets in the surrounding environment. The key performance indicator (KPI) requirements for WLAN sensing as defined by IEEE 802.11bf include: (i) a coverage range to be set for the successful detection of targets by WLAN sensing, where the maximum allowable range of the signal-to-noise ratio (SNR) between the sensing stations and the targets should be above a pre-defined threshold (conventionally taken as 10dB or 13dB); (ii) a field of view (FOV) and resolution range to be set to estimate the angle of the coverage area through which the STA performs sensing to find the minimum distance between two targets in the same direction, respectively; (iii) the latency and refresh rate to be defined to estimate the time to complete the related WLAN sensing process and the frequency for refreshing the sensing task, respectively; (iv) the ratio of the number of correct predictions to the number of all possible predictions. The prediction tasks can be: (a) gesture detection, where a pre-defined set of gestures and/or motions shall be identified; (b) presence detection; (c) a specific body activity detection, such as breathing; (d) real person detection, distinguishing human beings from other objects.

### 3.5. Edge Computing and the Tactile Internet

With the proliferation of the IoT for many sensing devices in AR/VR applications, these devices suffer from limitations regarding the data, resources for computing, and communications [87]. It has been estimated by Cisco that the data generated by machines, IoT devices, and people are likely to exceed by 500 zettabytes (ZB) [88]. Edge cloud computing is considered a solution to overcome the above-mentioned challenges. This also reduces latency for end users due to the capability of providing computational and storage resources at the edge of the radio access network (RAN). In order to satisfy the VR/AR ultra-low latency requirements, there should be a suitable reduction in communication distance, which can be achieved by multi-access mobile edge computing (MEC) by bringing the applications closer to the end users [89,90]. MEC acts as a key enabler of 5G technology due to its main functionalities of computation and storage at edge hosts located close to the radio access network nodes (e.g., gNodeBs in 5G) in a distributed manner. Due to the distributed nature of the network, MEC enables low-latency services with the requirement of a very low and bounded delay between the end user devices and the server hosting the application. Due to the challenges faced by the stringent requirements of AR/VR technologies, the 5G-enabled Tactile Internet (TI) with computation-intensive tasks on edge computing servers is considered as the most-reliable solution to obtain ultra-reliable and sub-millisecond latency communication [91].

The TI is an innovative tool used for AR/VR applications comprising sensors, actuators, robotics, computation components, and dedicated hardware (e.g., wearable devices) to empower tactile and haptic (e.g., touch, visual, and auditory) feedback in Internet-based applications [92]. The TI is assumed to be the next evolutionary phase of the Internet of Things (IoT) utilizing machine-to-machine (M2M) and human-to-machine (H2M) interactions to incorporate a wide range of application scenarios in the industrial, eHealthcare, education, and entertainment sectors. With the usage of 5G communications, the TI has turned into an evolutionary technology for the IoT and Society 5.0 by providing delay mitigation and enabling real-time interaction with haptic data over the Internet to address complex issues in our current society [93]. Three different layers of the TI, namely the master, network, and slave layers, should be analyzed carefully while integrating 5G with the TI for ultra-reliable low-latency applications. The master layer has an important role in the conversion of the human input or haptic feedback (from a communication perspective) into tactile data by using a human–system integration present at the layer. Then, the HSI transmits the tactile data created by suitable tactile encoding techniques over the controlled domain via the network layer. The controlled domain provides all the haptic feedback signals including audio/visual feedback signals to the master layer connected over the network domain via a two-way communication link. With its integration with 5G, low-latency communication is achieved by incurring a round-trip time (RTT) of less than 5ms, which will support haptic communication [94].

## 4. Trade-Offs among Performance Parameters

In this section, we describe several factors that are responsible for maintaining the performance parameter trade-offs in order to enable a low-latency and reliable wireless network for AR and VR applications. In Section 4.1, we discuss the contribution of an innovative and smart wireless network design towards a low-latency and reliable wireless network by balancing the trade-off of the latency measures with other performance factors. Section 4.2 presents a few ways to ensure a balanced trade-off between reliability and latency for the wireless scenario of AR/VR network design. Section 4.3 shows the importance of quality of experience (QoE) in affecting the latency to satisfy the low-latency requirements of AR/VR HMDs.

### 4.1. Design of 5G Wireless Network Architecture

**Figure 5:**
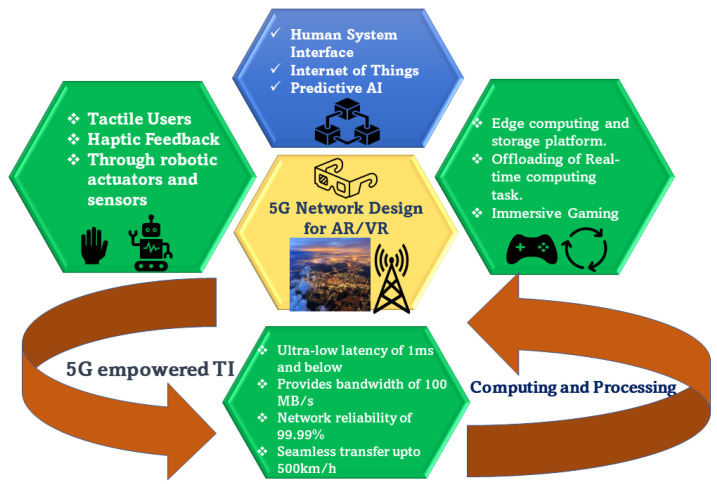
This diagram depicts the capabilities of a 5G-enabled network design that employs cutting-edge technologies such as edge computing and the Tactile Internet to enable low-latency communication for AR and VR use cases.

The design of a smart 5G wireless network, as shown in Figure 5, is an innovative solution to enable ultra-low-latency and reliable communications in emerging and near-real-time applications such as augmented and virtual reality, remote surgery, self-driving cars, and multi-player online gaming. For an interactive VR gaming arcade, a smart network design was shown in [95] to realize the performance of immersive VR gaming scenarios, characterized by reliable and minimal latency. This setup of an indoor VR gaming arcade requires very low latency for synchronization of the location and the interactions among a group of players. The HMDs of the players in [95] were equipped with multiple mmWave band access points (mmAP) connected to an edge computing network having an operational bandwidth of around 60GHz. The edge computing network consisted of multiple edge computing servers and a cache storage unit to offload the player’s tracking data and predict poses from a prediction window for every user’s HD frames obtained from their real-time tasks. This network design arrangement helps maintain a trade-off between communication latency and computation latency with efficient task offloading decisions built for efficient server placement and server selection. The need to design a cellular control plane for core networks to avoid the delay experienced by end-user applications arises. This led to the design of Neutrino, a cellular control plane [96] for providing an abstraction of reliable access to cellular services by connecting the IP backbone to the base stations while ensuring lower latency at the same time. By regularly updating the user state to the user’s data access established to the Internet or to other operator services, the cellular control plane overcomes the challenge of providing low latency and reliable access to the cellular core network by enabling connectivity sessions for each device. The control plane follows a consistency protocol to minimize service disruption under failures to verify if the devices are always able to receive read-your-writes consistency [97]. The latency of control plane had a direct impact on the data latency shown in the study [98], where the control functions contributing a 72.5–99.6 ms latency in session establishment can cause up to a 1.9ms delay in data access. The Neutrino test setup utilized two dual-socket servers running Ubuntu 18.04.3 with Kernel 4.15.0-74-generic, with 18 cores per socket and a total memory of 128 GB. Further optimizations were performed of Neutrino to satisfy the acceptable bandwidth trade-off for the cellular providers and to reduce the issues of increasing overhead in messages to profit from the latency benefits.

A UAV-aided wireless network design [99] can be utilized to reduce over-the-air delay through channel condition improvement techniques at both the transmitter and receiver by reducing the packet error probability to enable a reliable and low-latency communication channel in AR/VR. A remote VR gaming setup was presented in [100] using a low-latency edge rendering scheme. It utilizes two gaming laptops equipped with an Nvidia GTX 1070 GPU and Intel i7 7820HK CPU, with both connected using Gigabit Ethernet for low-latency communication. This scheme allows the execution of a VR game by off-loading the information from an end user device to a cloud edge server to render the game based on the feedback obtained from the headset and the controller network. This setup is able to achieve an end-to-end latency of 30 ms with a bit rate of 20 Mbps for the stereo 1080p30 format. VLC, emerging as a powerful interface solution for 6G, has the potential to boost the transmission rate by utilizing a micro-LED-based photodetector [101] in AR/VR-based HMDs. This detector uses a receiver fabricated on a Si substrate that can achieve a transmission rate of more than 10 Gbps with visible light communication. The Si substrate LED-based transmitter with multiple superlattice interlayers [102] also possesses the capability to increase the transmission rate beyond 24 Gbps. A free-space optical stealth communication system was introduced in [103] to accelerate the data rate and secure the user’s information in wireless communication networks. Photonics technology coupled with the W band also has the capability to improve the transmission rate by 47.45 Gbps for signal delivery over a 10 km single-mode fiber-28 (SMF-28) and 4.6 km wireless free-space link [104]. A latency-driven MEC-VR design was highlighted in [105] to locally render at a high refresh rate, and the evaluation was performed at an MEC node. A remote server was used by the MEC-VR users to generate larger scenes compared to the client’s FoV as the size of the margin area of the FoV was adaptively adjusted for the prediction of the next head movements according to the observed system latency. The MEC server [106] is considered as a promising solution to achieve ultra-low-latency communication by reducing the additional time required for collecting and analyzing data when placed closer to the users at the network’s edge.

### 4.2. Reliability

The reliability of the traffic behavior of information or video frames is being questioned in the wireless scenario of AR/VR network design due to the temporary outages occurring from the impairments in the signal-to-interference-plus-noise ratio (SINR). However, increasing the reliability always results in additional delay as re-transmission to send the rectified frames at the physical layer by using the parity increases the latency. The most-important reliability aspect in 5G to ensure a smooth and immersive AR/VR experience is obtaining an ultra-high success rate of tracking message signaling with a maximum packet error rate (PER) of $10^{-5}$. Hence, these packets have to be delivered with ultra-high reliability to ensure a smooth VR service. Based on these requirements, a scheduling optimization framework was shown in [95] to satisfy the trade-offs between stringent latency and reliability constraints for an immersive VR gaming experience in the case of an unsuccessful frame delivery. The HD frames corresponding to the user’s upcoming movement and head rotation are rendered by a joint proactive computing and caching scheme following different priority levels for real-time computing. To allocate the mmWave transmission resources to users by prioritizing their requests with tight latency deadlines, a matching algorithm based on the deferred acceptance (DA) matching [107] was considered for matching preferences to meet the requirements for the reliability and latency constraints.

### 4.3. Quality of Experience

Several studies have been conducted to analyze the quality of experience (QoE) [108], which implies the detailed subjective experience of the effects observed during task completion with the use of a VR simulator. It has been found that the latency has a huge impact on the QoE from the subjective studies performed on a log-loading task [109] of a forestry crane with a VR simulator. These subjective studies were performed on the VR system by adding a controlled delay to the display update and the joystick update. The added delays observed from the task were the controlled delays ranging from 0 to 30ms for the display update and from 0 to 800ms for the hand controller when referred to the delays in the crane control interface. The simulator used for performing the task was VR goggles (Oculus Rift) [110], providing stitched stereo camera views, joysticks for controlling the crane, and a simulation environment required for lifting logs onto the truck. The delays obtained by the simulator software developer baseline system for the VR simulator were estimated to be 25ms in the screen update based on the movement of the head for rendering and about 80ms for the motion of the joysticks to the visual feedback on the screen. No significant delay effects were observed in the task performance in the joystick delay study within a range of 200 ms. In addition to delays in the task, the delay range was recorded as less than 30–35 ms for the latency of the joysticks or hand controllers. With the evolution of the high speed of the fifth-generation communication technology, Multi-access MEC technology has been proven as a potential tool to satisfy the ultra-low latency requirements of 360° video delivery to promote users’ QoE [111].

## 5. Discussion and Future Scope

With the advancement of 5G technology, AR/VR requires significant resources for real-time processing, i.e., rendering, vision, and physics engines, at end user devices to maintain its ubiquitous nature across heterogeneous networks, including 4G, 5G, and WiFi. Usually, in mission-critical networks, the delivery of small information packets (32 to 200 bytes) within a latency of 1 ms [112] results in a huge computation delay with the traversal of each packet undergoing the procedure of processing, queuing, and transmitting through multiple routers, where each router contributes 10 ms of computation delay [14]. However, the adoption of high-end hardware and processing approaches such as the Multi-Path Transmission Control Protocol (MPTCP) [113] to reduce the computational complexity and transmission instability can only reduce the latency by 5–6 ms for time-sensitive AR/VR applications. These challenges highlight the need for innovative solutions to enable ultra-low-latency and reliable transmission for real-time updating across 5GB networks [114]. Though existing networks are able to offer significant enhancements for rich content delivery, they cannot meet the demand for up to a 5.2Gbps data rate per user and an end-to-end RTT of 1ms [115]. The requirement of achieving <1 ms for the transmission of information from the total end-to-end RTT on the networks is still a challenging task as the latency between end users continues to increase with distance. As traditional communication systems focusing mostly on error-free transmission cannot satisfy the transmission rate requirements of AR/VR due to the limitation of Shannon’s capacity, we require a semantic communication approach to achieve end-to-end latency through a novel transmission protocol or algorithm. Considering the above-mentioned requirements, we propose a semantic-communication-based delay-aware algorithm, which adopts an intelligent routing protocol to extract the semantic level features such as (i) the selection of the most-optimal or the shortest path to ensure the instant packet delivery of prioritized information and (ii) the accurate prediction of the routing decision by evaluating the subflow’s delivery performance in sending prioritized content with the lowest latency. With the motivation to improve the traffic capacity requirement, which plays an integral role in the 6G era, our proposed approach can be capable of identifying/predicting the accurate route for the transmission of selected information while maintaining a low-latency communication [116]. With the integration of AI/ML techniques similar to a few existing wireless B5G networks [117,118] to increase the transmission rate beyond Shannon’s capacity, we introduced an AI/ML integrated learning algorithm to enable the proposed end-to-end semantic communication approach.

**Figure 6:**
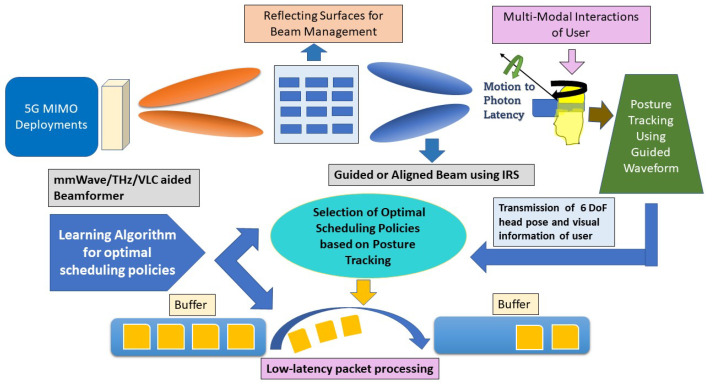
The block diagram depicts a futuristic approach that employs several advanced 5G/5GB technologies for instant packet delivery of prioritized information in AR/VR to ensure ultra-low-latency transmission. The 5GB beamforming technology is introduced by utilizing a holographic MIMO to produce guided beams to track the multi-modal sensory information of the user. Then, the tracking information will undergo a learning algorithm based on the scenario to decide the optimal route for the timely delivery of the buffer packets to reduce the latency.

To implement delay-aware semantic communication involving a proactive transmission strategy of data packets for high delivery rates of video frames (VFs), interference-aware directional beamforming technology is an essential tool to generate highly optimized directional beams. Moreover, this approach requires some preprocessing steps to overcome the bandwidth limitations when handling packet overloading [119], which causes complications in the external sensing environment. By utilizing optimized directional beams as the preprocessing steps followed by the transmission strategy, the selection of a suitable spectrum becomes necessary to handle the recent bandwidth congestion. To overcome the problem of spectrum congestion due the explosive growth of data traffic generated by mobile devices and users, a new license-free optical spectrum, known as visible light communication (VLC), can be envisioned [120,121]. VLC, known for its secure transmission compared to traditional radio frequency (RF) communications, can be enabled through a setup of optical wireless interfaces at the HMDs. Hence, the use of VLC can reduce latency by its capability to simultaneously enhance illumination and communication to the users through a single device. MIMO structures [20] are required to ensure higher reliability and data throughput to provide a reduced latency and increased robustness to the VLC system. We can utilize a holographic MIMO [122] as a part of this novel beamforming technology to enable the evolution of wireless networks by allowing pervasive communications between humans and AR/VR devices. Such MIMO structures designed for 5GB have an intelligent and software-reconfigurable paradigm responsible for fulfilling the visions of ultra-low-latency communications, high throughput, and high connectivity. This holographic MIMO structure, consisting of an intelligent reflecting surface (IRS), known to increase transmission efficiency and generate radiation in the desired direction, is highly recommended to reduce the interference arising from the simultaneous transmission of packets. Then, tracking is performed by the mmWave/THz/VLC bands through the highly focused directional beams of the proposed beamforming technology to exploit user information (6 DoF), which includes location (up to 1 mm) and orientation (up to 0.5°). As the position and motion of the user over time are unknown to the radio access network (RAN) due to the dynamic nature of the headsets and wireless channel, the beam faces difficulties in adjusting its alignment according to the posture of the user through the HMD. As several AI-/ML-based techniques [123,124] relying mostly on synthetic datasets for training and testing for performance verification produce unrealistic results due to the huge difference between the simulation settings and real network environments, we propose a learning algorithm for interacting with the dynamic and random virtual environment where the information packets can be successfully transmitted using an optimal scheduling policy. Furthermore, an optimal transmission queuing strategy can be utilized for the user’s information based on the tracking results for their timely delivery, as presented in Figure 6. This figure shows the usage of several advanced technologies such as the deployment of a MIMO-based mmWave beamformer coupled with an intelligent reflecting surface (IRS) to produce guided beams to the users for low-latency packet processing. We would like to highlights the requirement for a novel learning approach of an optimal routing protocol or scheduling policy through beam alignment to meet the ultra-low-latency requirements in AR/VR. There are different techniques involved in obtaining an effective scheduling policy based on the AR/VR applications to avoid video buffering. The steps involved in the learning algorithm for different scenarios are as follows:

- We divide the environment into simple scenarios (watching movies, attending virtual meetings, etc.) and hard scenarios (playing games, virtual training, etc.) based on the movement of the user’s HMD.
- For simple scenarios, we can consider reinforcement learning [125], where the the environment is defined by utilizing highly focused directional beams to track the posture of the HMD. Then, the action is based on the selection of the optimal beam for data transmission by learning (exploiting) the user’s environment, i.e., the posture tracking of the user. Finally, the reward is decided by the action, which is an optimal scheduling policy that minimizes the delay during data delivery.
- For hard scenarios, we can apply the combination of reinforcement learning and supervised learning, known as imitation learning (IL) [126], to tackle the rapid loss of the buffer packets as a result of frequent multi-modal interactions in such scenarios. This includes a preprocessing step, which involves generating an expert dataset through supervised training based on the possible outcome of the guided beams given that the pose of tracking (feedback) is known to the user. The action is based on learning a policy through behavior cloning [127] by mapping the multi-sensory information with the respective optimal beam based on the pre-trained dataset. The reward is then determined from the action to select the best scheduling policy for a low-latency data delivery. Hence, the goal of achieving the trade-off between beam alignment and data transmission in such scenarios for low-latency communication in AR/VR is made possible by IL.

**Figure 7:**
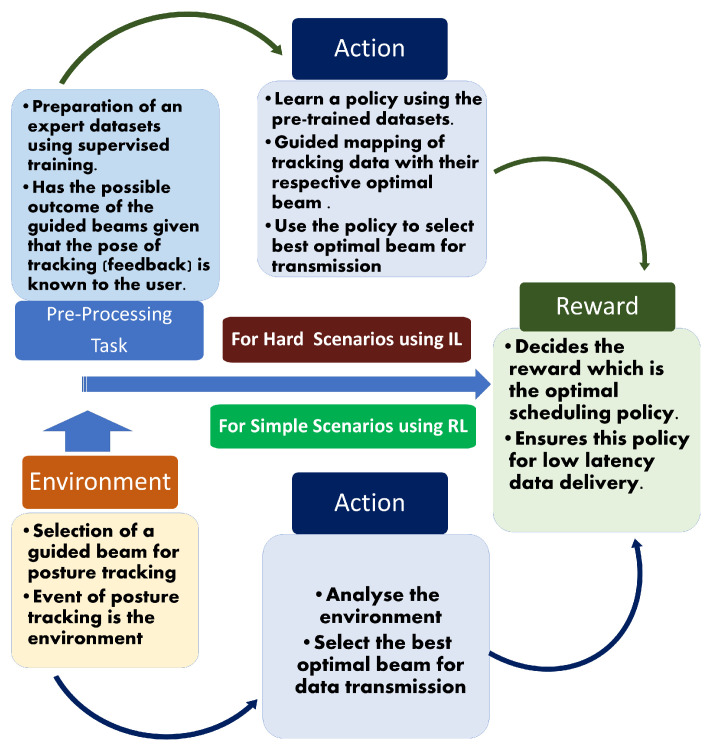
This diagram describes different methodologies involved in the proposed learning algorithm to decide the optimal scheduling policy for low-latency data delivery. Two main categories of scenarios were assumed, and reinforcement learning (RL), as well as imitation learning (IL) are tailored to them accordingly.

The algorithms as shown in Figure 7 will help to decide the priority-based transmission scheduling policy based on the user’s behavior determined by multi-modal interactions. Multi-modal interactions, involving unimodal inputs such as visual-based, sensor-based, kinetic-based, and audio-based inputs, are widely analyzed based on human behavior for immersive communication in a virtual environment. The TI can be considered with 5G/5GB technology for the transmission of multi-modal sensory information to increase the perceptual performance. However, the latency, transmission rate, and sampling rate are affected due to their asynchronous nature, which leads to the requirement of an effective multiplexing scheme to integrate these different modalities. The investigation of suitable AI/ML techniques to improve the timeliness of data delivery should be performed to deploy various multiplexing schemes and facilitate the transmission over the packet-switched networks in AR/VR wireless TI networks. Even the advancement of such ML techniques can be used to enable highly accurate predictions of channels, traffic, states, and other key performance indicators in an URLLC wireless virtual environment [128].

## 6. Conclusions

This article provided insights into the various contributions of technological advancements in 5G and B5G networks to enable ultra-low latency for a high-quality immersive experience in AR/VR technologies. The main categories of AR/VR applications along with their benefits in each category were discussed. The major requirements of the powerful technologies in different emerging AR/VR use cases were highlighted from the hardware and software perspectives to enable a low-latency AR/VR system. Several factors responsible for maintaining the trade-offs of the performance parameters in order to enable a low-latency and reliable wireless network were presented. An innovative wireless network design integrated with ground-breaking technologies such as WiFi 7, the Tactile Internet, and edge computing were shown as the key enablers of 5G and the IoT in AR/VR applications. The usage of these technologies was highlighted as the most-powerful tools to perform intensive tasks while operating through an ultra-reliable and sub-millisecond-latency communication to enable a high-connectivity ecosystem. Future directions with a focus on high update rates for package delivery for low latency transmission were explored in this article. An optimal transmission queuing strategy was described with the use of several advanced technologies, such as the deployment of MIMO-based mmWave beamformers and intelligent reflecting surfaces for the timely delivery of the user’s tracking information. Moreover, the combination of VLC with holomorphic MIMO was presented as a potential solution to enable high reliability and data throughput in an AR/VR wireless architecture. An accurate route for transmission can be obtained by using this optimal strategy by exploring the semantic level of features by selecting or predicting the shortest path for high delivery of video frames. Visionary solutions, including the introduction of learning algorithms using RL and IL based on different scenarios, were described to achieve an optimal selection of the best scheduling policy for an evolved and extremely immersive AR/VR experience.

## Figures and Tables

**Table 1:** The acronyms used in the paper with their respective meanings.

Acronym	Definition	Acronym	Definition
5GB	5G and Beyond	MEMS	Micro-electromechanical systems
AR	Augmented reality	MTP	Motion-to-photon
AV	Autonomous vehicles	NCC	Normalized cross-correlation
DNN	Deep neural network	NLOS	Non-line-of-sight
DoF	Degree of freedom	OFDM	Orthogonal frequency-division multiplexing
EKF	Extended Kalman filter	OST	Optical see-through
eMBB	Enhanced Mobile Broadband	PDCP	Packet Data Convergence Protocol
FOV	Field of view	PER	Packet error rate
fps	Frames per second	QoE	Quality of experience
HARQ	Hybrid automatic repeat request	QoS	Quality of service
HDD	Heads down display	RTT	Round-trip time
HDR	High dynamic range	SOC	System-on-a-chip
HMD	Head-mounted display	TI	Tactile Internet
HUD	Heads up display	URLLC	Ultra-reliable low-latency communication
IoT	Internet of Things	UWB	Ultra-wideband
IMU	Inertial measurement unit	VIO	Visual–inertial odometry
KPI	Key performance indicator	VLC	Visible-light communication
MAC	Multiply–accumulate	VR	Virtual reality
MAR	Mobile augmented reality	WLAN	Wireless local area network
MEC	Mobile edge computing		

## Data Availability

Not applicablet.
